# Correlation between Androgen Receptor Expression in Luminal B (HER–2 Negative) Breast Cancer and Disease Outcomes

**DOI:** 10.3390/jpm12121988

**Published:** 2022-12-01

**Authors:** Fan Yang, Jiayi Li, Hong Zhang, Shuang Zhang, Jingming Ye, Yuanjia Cheng, Qian Liu, Ling Xin, Hongyu Xiang, Yinhua Liu, Xuening Duan, Ling Xu

**Affiliations:** 1 Breast Disease Center, Peking University First Hospital, Beijing 100034, China; 2 Medical Cosmetology, Beijing Jishuitan Hospital, Beijing 100035, China; 3 Department of Pathology, Peking University First Hospital, Beijing 100034, China

**Keywords:** breast cancer, neoadjuvant therapy, androgen receptor, AR/ER, Ki67

## Abstract

(1) Background: Hormone receptor positive breast cancer is a subtype of breast cancer with relatively good prognosis, but luminal B (HER–2 negative) breast cancer has a higher risk of recurrence and metastasis. Patients with endocrine therapy resistance and chemotherapy insensitivity have poor prognosis. Androgen receptor (AR) is widely expressed in breast cancer, but there is no clear conclusion about its function and correlation with prognosis in luminal B breast cancer. Further research is needed to reveal the role of AR in luminal B (HER–2 negative) breast cancer. (2) Methods: Retrospectively analyzed patients with early–stage luminal B breast cancer. The correlation between AR and its associated indexes with long–term survival was determined. (3) Results: A total of 985 patients were included with 143 treated by neoadjuvant therapy. Of these, 83.5% of the patients had AR expression ≥65%. High AR expression was associated with good disease–free survival (DFS) and overall survival (OS). In the neoadjuvant population, AR/estrogen receptor (ER) > 1.06 and residual tumor Ki67 > 23% had significantly worse DFS. (4) Conclusion: Low AR (<65%) expression is associated with poor prognosis in luminal B (HER–2 negative) breast cancer patients. High AR/ER and residual tumor Ki67 were associated with poor DFS in neoadjuvant group with a cutoff value of AR/ER > 1.06 and residual tumor Ki67 > 23%.

## 1. Introduction

Hormone receptor (HR)–positive breast cancers account for about 75% of all breast cancers [1,2,3]. They can be classified as luminal A or B (HER–2 negative) based on their expression of the proliferation index Ki67 and progesterone receptor. Luminal A breast cancers respond well to endocrine treatment efficacy and have good prognosis. Luminal B (HER–2 negative) breast cancers have lower levels of HR expression and higher proliferation indexes than luminal A as well as a higher risk of recurrence and metastasis. Based on these characteristics, the comprehensive treatment plan for luminal B (HER–2 negative) breast cancers includes surgery, radiotherapy, endocrine therapy, and chemotherapy. Most luminal B breast cancers are sensitive to endocrine therapy; however, some patients develop primary or secondary resistance to endocrine therapy. Currently, the prediction and reversal of endocrine resistance and the search for novel therapeutic agents are popular research topics. Some luminal B breast cancers are less sensitive to chemotherapy and have a poor prognosis even with chemotherapy. There is a lack of sensitive indicators to predict sensitivity to chemotherapy and whether patients can benefit from specific chemotherapy regimens. This suggests that large–scale studies are needed to review the factors associated with the treatment and outcomes of luminal B (HER–2 negative) breast cancers and to search for novel predictors.

The systemic treatment of breast cancer can be classified as neoadjuvant or adjuvant therapy, depending on its timing. Neoadjuvant therapy plays a vital role in the treatment of breast cancer, not only to reduce the extent of surgery, and obtain information on treatment responsiveness and prognosis, but also to adjust subsequent treatments based on the treatment response to improve the survival of patients in non–pathological complete response (non–pCR) patients. In HER–2–overexpressing breast cancer and triple–negative breast cancer (TNBC) patients, the rate of pCR can reach 40–50% with neoadjuvant treatment, and the long–term survival of patients achieving pCR is significantly better. Luminal B (HER–2 negative) breast cancer lacks HER–2 targets, and the current standard neoadjuvant treatment regimen is chemotherapy; neoadjuvant endocrine therapy is still being investigated. Luminal B (HER–2 negative) breast cancer has poor sensitivity to neoadjuvant therapy, with a pCR rate of 5–10%, and the predictive value of pCR for long–term prognosis is not as high as that for HER–2 positive breast cancer and TNBC. Therefore, there is a need to find novel indicators to predict the efficacy of neoadjuvant therapy and outcomes for luminal B (HER–2 negative) breast cancers. The literature suggests that Ki–67 can be used to evaluate the efficacy of neoadjuvant endocrine therapy in patients with luminal B (HER–2 negative) breast cancer. However, the cutoff values and predictive sensitivities in different reports vary widely, and its predictive ability lacks validation in neoadjuvant chemotherapy.

The androgen receptor (AR) is widespread across various breast cancer subtypes, and over 70% of HR–positive breast cancers express AR. Most retrospective studies have found that positive AR expression is associated with a better long–term prognosis of breast cancer [4,5]. However, the correlation between its expression and the efficacy of neoadjuvant therapy is inconsistent across breast cancer subtypes. In TNBC, AR positivity is associated with a poor response to neoadjuvant therapy, whereas in HER–2–positive breast cancer, AR expression suggests a good response to neoadjuvant therapy. Studies on the correlation between AR and prognosis and its function in luminal B (HER–2 negative) breast cancer were contradictory. AR may play an oncogenic role, as AR agonists have the potential to reverse endocrine resistance [6]. However, high AR expression may be associated with endocrine resistance, as patients with ER–positive breast cancer with high ratios of AR expression to estrogen receptor (ER) expression (AR/ER) have poorer disease–free survival (DFS) [7]. In these studies, the stage and status of cancer differ, and the ranges of AR, AR/ER, and ratio of AR expression to PR expression (AR/PR) differ as well, which could lead to opposite conclusions. In HER2–positive breast cancer, the expression of AR is elevated, regardless of the status of hormone receptor. It may be related to the positive feedback cycle between AR and HER2. The relationship between AR expression and prognosis in luminal B HER–2 positive breast cancer is not clear, and the conclusions of different studies are inconsistent [8,9]. Therefore, a larger sample size and more in–depth mechanistic studies are needed to investigate the effects of AR on the development and prognosis of breast cancer.

In this single–center study, cases of luminal B (HER–2 negative) breast cancer at the Peking University First Hospital were retrospectively analyzed to investigate the correlation between AR and survival and neoadjuvant treatment outcomes in luminal B (HER–2 negative) breast cancer, and to determine AR cutoff values with clinical value.

## 2. Materials and Methods

### 2.1. Study Participants

**Figure 1:**
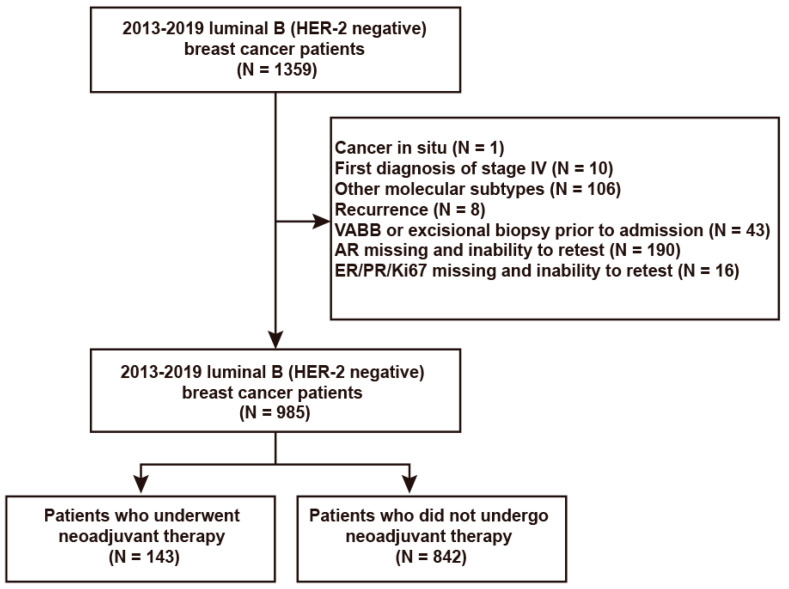
Flowchart of inclusion of luminal B (HER–2 negative) breast cancer patients.

Patients with early–stage luminal B (HER–2 negative) breast cancer admitted to the Peking University First Hospital Breast Center between January 2013 and December 2019 were retrospectively analyzed. The inclusion criteria were patients with luminal B (HER–2 negative) breast cancer diagnosed using core needle biopsy (CNB) and treated by surgery at our hospital. The exclusion criteria were patients who had the following: (1) first diagnosis of stage IV breast cancer; (2) AR expression level missing from pathology report and inability to retest; and (3) underwent excisional biopsy or ultrasound–guided vacuum–assisted breast biopsy (VABB) prior to admission (Figure 1). The hospitalization information of patients was obtained by reviewing the case database and followed up once a year primarily by telephone and supplemented by reviewing outpatient and inpatient data. Data on re–examinations, current treatment, recurrence, and death were collected.

### 2.2. Specimen Preparation and Staining

Preparation of CNB and surgically resected specimens was as described in the 2007 American Society of Clinical Oncology and the College of American Pathologists (ASCO/CAP) guidelines [10]. Specimens were fixed promptly after isolation (no more than 1 h of cold ischemia). Surgical specimens were cut open into 5 mm page–like sections and separated by gauze. Subsequently, they were fixed in 4% neutral buffered formaldehyde for 6–48 h, embedded in paraffin, processed into sections 4 μm in thickness, and stained with hematoxylin–eosin (HE). The slides were read by two Peking University First Hospital pathologists for pathological diagnosis of histological type and grading. Histological scoring and grading were performed based on glandular duct formation, size and shape of cell nuclei, chromatin heterogeneity, and mitotic figures in accordance with the criteria for the Nottingham combined histologic grade [11]. The anatomical staging was performed in accordance with the 8th edition of the American Joint Committee on Cancer (AJCC) breast cancer staging system [12].

### 2.3. Immunohistochemistry (IHC) and Fluorescent In Situ Hybridization (FISH)

IHC for ER, PR, HER–2, AR, and Ki67 was performed for all included cases. In this study, ER (antibody: 1D5, Dako), PR (antibody: 636, Dako), AR (antibody: EP120, GBI), and Ki67 (antibody: MIB1, Dako) staining was performed using a fully automated IHC stainer (Dako Autostainer Link 48), and HER–2 (antibody: 4B5, Ventana) staining was performed using a fully automated IHC stainer (Ventana, Bench–mark X). In this study, ER, PR, and HER–2 were tested in accordance with the 2010 ASCO/CAP Guideline Recommendations for Immunohistochemical Testing of Estrogen and Progesterone Receptors (ER/PgR) in Breast Cancer [13] and the 2013 ASCO–CAP HER–2 Test Guideline Recommendations [14]. The proportion and intensity of positive staining of tumor cells were recorded as H–score. Based on the H–score (0–300) = staining intensity (0, 1, 2, 3) × percentage of positive cells, specimens were classified into one of four grades: negative (−): 0–50, weakly positive (+): 51–100, moderately positive (++): 102–200, and strongly positive (+++): 201–300. ER and PR positivity was defined as ≥1% of tumor cells with positive nuclear staining, and negativity was defined as <1% of tumor cells with positive nuclear staining. According to the 2013 ASCO–CAP HER–2 Test Guideline Recommendations [14], IHC +++ was considered positive, and IHC − or + was considered negative. For IHC ++, FISH was performed to test for gene amplification. FISH testing was performed using a dual–probe kit (LBP, Guangzhou, China). The results were interpreted as specified in the 2013 ASCO–CAP HER–2 Test Guideline Recommendations [15]. In the region, a ratio of HER–2 to the centromeric region of chromosome 17 (CEP17) (HER–2/CEP17) < 2.0 was obtained by counting 20 or more cells in the region, and a mean HER–2 gene copy number < 4.0 was considered negative, and HER–2/CEP17 ≥ 2.0 and mean HER–2 gene copy number ≥ 4.0 or HER–2/CEP17 < 2.0 and mean HER–2 gene copy number ≥ 6.0 were considered positive. Ki67 positivity was determined by calculating the percentage based on the number of cell nuclei stained, and ≥ 14% was defined as positive. The molecular typing of breast cancer was estimated based on the immunohistochemical expression of ER, PR, HER–2, and Ki67 in accordance with the St. Gallen International Expert Consensus guidelines [16]. Luminal B (HER–2 negative) was defined as positive ER expression, low PR expression or high Ki67 expression, and negative HER–2 expression. AR expression level was described as the proportion and intensity of nuclear staining.

### 2.4. Neoadjuvant Treatment Regimen and Evaluation Criteria

Among the included patients undergoing neoadjuvant therapy, neoadjuvant chemotherapy and neoadjuvant endocrine therapy were allowed. The first choice for the neoadjuvant chemotherapy regimen was 6–8 cycles of taxanes + anthracycline (TA), with the specific drugs being ① T: docetaxel 75 mg/m^2^ intravenous infusion once every 3 weeks or albumin–bound paclitaxel 200–260 mg/m^2^ intravenous infusion once every 3 weeks; ② A: pirarubicin 50 mg/m^2^ intravenous infusion once every 3 weeks. Response evaluation was performed every two cycles in accordance with the Response Evaluation Criteria in Solid Tumors (RECIST) 1.1, and patients underwent surgical treatment after completing neoadjuvant therapy. The pathological response was evaluated using the Miller–Payne system. According to the recommendations of the Collaborative Trials in Neoadjuvant Breast Cancer (CTNeoBC) international working group, pCR was defined as the absence of invasive cancer in the breast and axillary nodes irrespective of ductal carcinoma in situ (ypT0/is ypN0) [17]. The decision to administer adjuvant chemotherapy was made based on the postoperative response to neoadjuvant therapy and the risk of recurrence. Endocrine therapy was administered for at least 5 years.

### 2.5. Statistical Analysis

For comparisons of clinicopathological characteristics between groups, the *t*–test for independent samples was used for continuous variables, Pearson’s χ^2^ test or Fisher’s exact probability test was used for categorical variables, and the Mann–Whitney U test was used for ranked variables. The odds ratio (OR) for a pathological response was calculated using a binary logistic regression model. The hazard ratio (HR) for long–term survival was calculated using Cox analysis. In both binary logistic regression and Cox analysis, continuous variables were analyzed by groups according to clinical practice and experience. DFS was defined as the time from the surgery date to recurrence and metastasis, death, or last follow–up. Overall survival (OS) was defined as the time from the surgery date to death or last follow–up. DFS/OS was analyzed using the Kaplan–Meier method, and differences between subgroups were tested using the log–rank test. Diagnostic tests were analyzed using ROC curve analysis. The selection of the best threshold is based on the boundary point when the Youden index is the maximum value. Differences with *p* < 0.05 were considered statistically significant. All tests were two–sided. All statistical analyses were performed using the IBM SPSS Statistics (Version 26.0; IBM Corp., New York, NY, USA).

## 3. Results

### 3.1. Patient Characteristics

A total of 985 patients, including 978 women and 7 men with a median age of 56 years, were included in the study. Patients were divided into neoadjuvant and non–neoadjuvant therapy groups based on the treatment modality, with 143 patients (14.5% of the total population) in the neoadjuvant therapy group and 842 patients (85.5% of the total population) in the non–neoadjuvant therapy group. Most patients in the neoadjuvant therapy group (88.1%) underwent neoadjuvant chemotherapy containing anthracycline and taxanes, and nine (6.3%) underwent neoadjuvant endocrine therapy. Seven patients achieved pCR for a pCR rate of 4.9%. The median follow–up time was 42 months (range: 4–101 months), with 37 DFS events and 10 OS events in the neoadjuvant therapy group and 46 DFS events and 19 OS events in the non–neoadjuvant therapy group during follow–up. The baseline characteristics of the overall population were shown in Table 1

Compared to patients in the non–neoadjuvant therapy group, patients in the neoadjuvant therapy group were mostly young and non–menopausal, in late clinical stage, and had a high histological grade, low ER, PR, and AR expression levels, and high Ki67 expression levels; the differences between the two groups were statistically significant. There were no statistically significant differences in body mass index (BMI) or HER–2 expression levels between the two groups.

Eleven patients (7.7%) in the neoadjuvant therapy group were AR–negative before treatment, 39 (27.3%) had AR expression levels of 1–64%, 21 (14.7%) had AR expression levels of 65–89%, and 72 (50.3%) had AR expression levels above 90%. Eleven patients (1.3%) in the non–neoadjuvant therapy group were AR–negative before treatment, 101 (12.0%) had AR expression levels of 1–64%, 196 (23.3%) had AR expression levels of 65–89%, and 534 (63.4%) had AR expression levels above 90%; the differences between the two groups were statistically significant (*p* = 0.032).

AR/ER and AR/PR were calculated as AR expression level/ER expression level and AR expression level/PR expression level, respectively. In cases of PR negativity, the AR expression level was used as AR/PR. Overall, 14.7% of patients had AR/ER > 1.00 and 55.1% had AR/PR > 1.00; the difference between the two groups was not statistically significant (AR/ER *p* = 0.809; AR/PR *p* = 0.347).

Ki67 expression before treatment was higher than 30% in 95 patients (66.4%) in the neoadjuvant therapy group and 207 patients (24.6%) in the non–neoadjuvant therapy group; the difference between the two groups was statistically significant (*p* = 0.003).

Among the non–pCR patients in the neoadjuvant therapy group, 48 patients (33.6%) had a residual tumor Ki67 over 20%. The ΔKi67 was calculated as ΔKi67 = residual tumor Ki67 − CNB Ki67, with “substantial decrease” defined as a decrease of at least 40%, “moderate decrease” defined as a decrease of 20–39%, “small decrease/no change” defined as a decrease of 0–19%, and “elevation” defined as a higher residual tumor Ki67 than CNB Ki67. A total of 31 patients (21.7%) exhibited a substantial decrease, and 15 patients (10.5%) exhibited elevation of Ki67 levels compared to preoperative levels. Details of the neoadjuvant therapy group are summarized in Table 2.

### 3.2. Prognosis–Related Risk Factors in the Overall Luminal B (HER–2 Negative) Breast Cancer Population

A multifactorial survival analysis was performed on the overall luminal B (HER–2 negative) breast cancer population using backward regression. Age, baseline T–stage, baseline N–stage, histological grade, baseline ER, PR, HER–2, Ki67, AR, AR/ER, AR/PR levels, and whether neoadjuvant therapy was administered as the factors initially included. A total of seven steps were performed, with age, baseline T–stage, baseline N–stage, histological grade, baseline AR, and whether neoadjuvant therapy was administered as the factors included in the end. The results of steps 1 and 7 are presented in Table 3. Age above 40 years and AR expression ≥90% were protective factors for DFS events; late T stage, axillary lymph node metastasis, and high histological grade were risk factors for DFS events; and ER, PR, HER–2, Ki67, AR/ER, and AR/PR were not significantly associated with DFS.

OS multifactorial survival analysis was also performed using the same backward regression with the same included factors as for DFS and a total of 8 steps. The factors included in the end were baseline T stage, baseline N stage, histological grade, CNB HER–2, and CNB AR. The results of steps 1 and 8 are presented in Table 4. AR expression ≥ 90% and low HER–2 expression were protective factors for OS events. Late T stage, axillary lymph node metastasis, and histological grade G3 were risk factors for OS events.

### 3.3. Prognosis–Related AR Cutoff Values in the Overall Luminal B (HER–2 Negative) Breast Cancer Population

**Figure 2:**
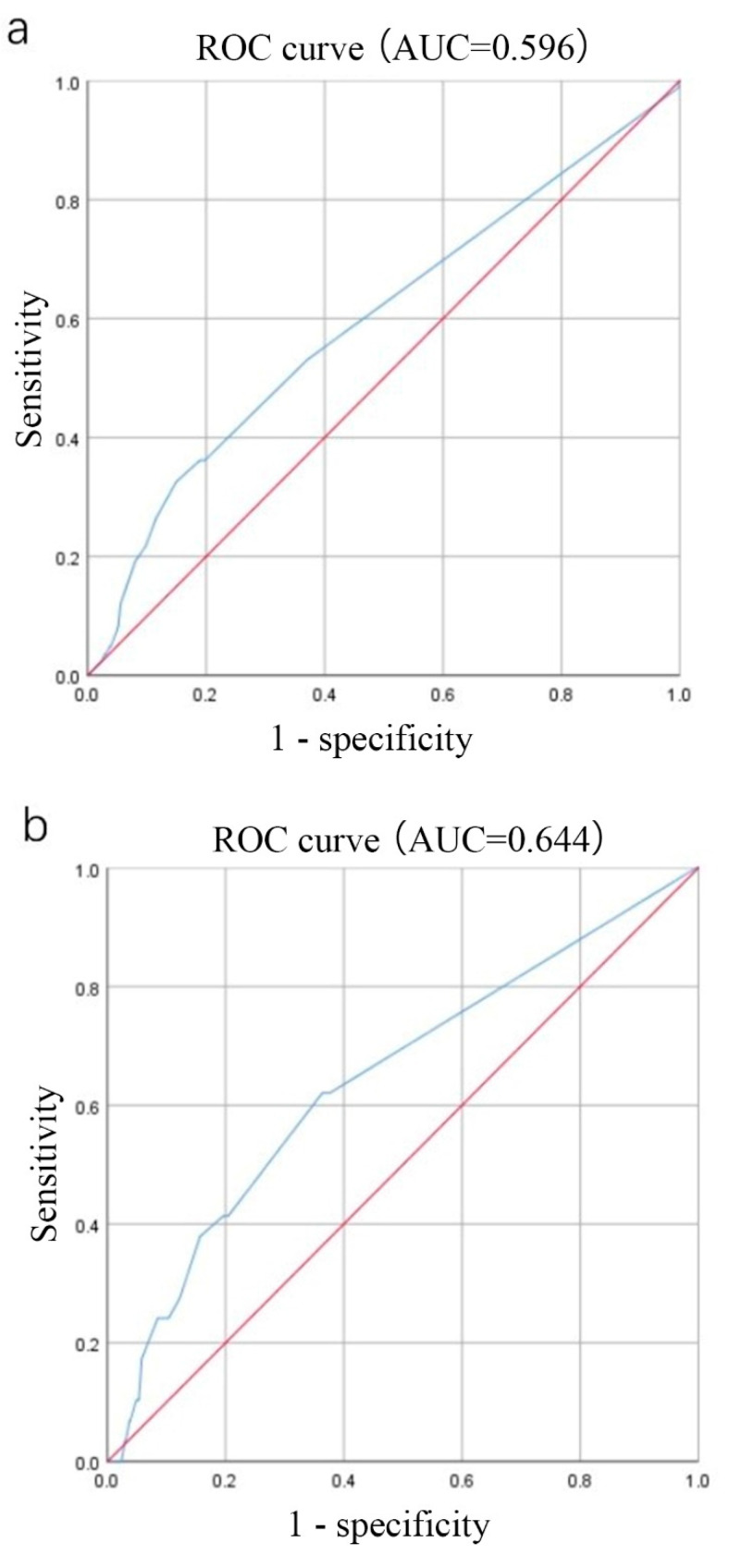

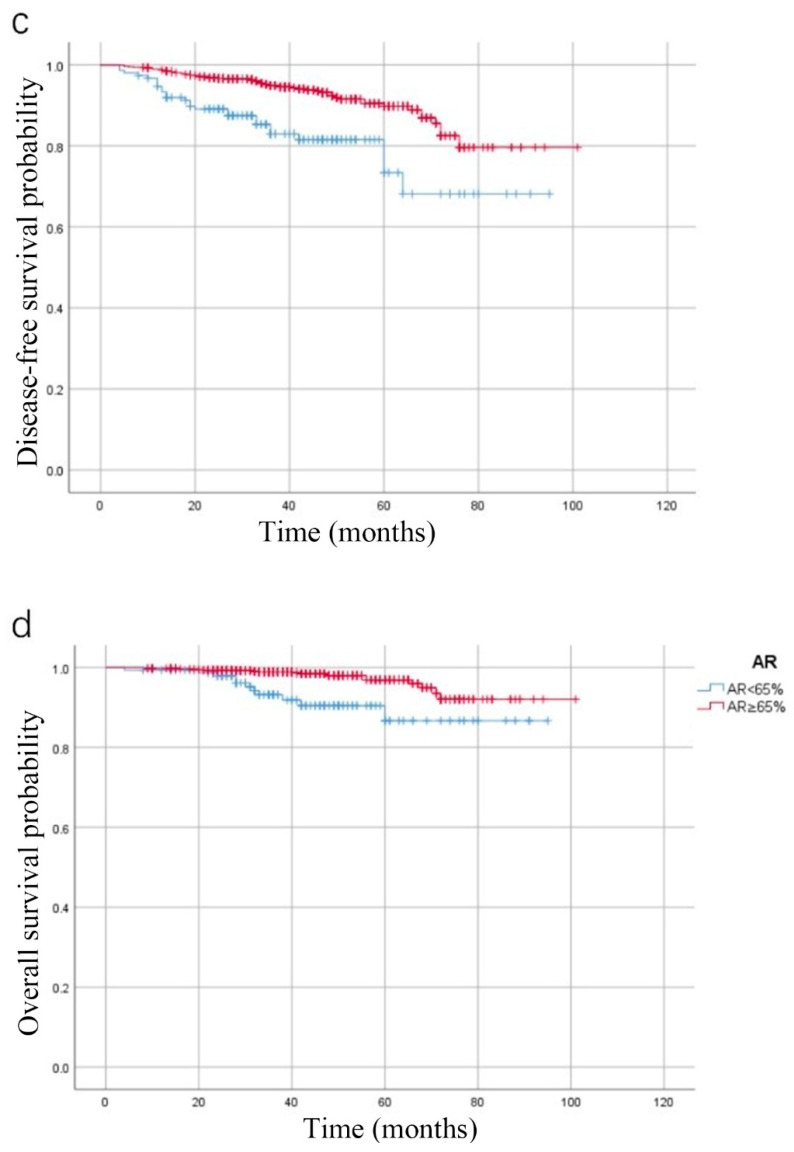
Prognosis of AR and early–stage luminal B breast cancer. (**a**) ROC curve of AR for predicting recurrent metastatic events; (**b**) ROC curve of AR for predicting mortality events; (**c**) Kaplan–Meier analysis of DFS; (**d**) Kaplan–Meier analysis of OS. AR, androgen receptor; DFS, disease–free survival; OS, overall survival.

In this study, the AR cutoff value was selected as 90% based on clinical experience. Multifactorial survival analysis suggested that AR ≥ 90% was a protective factor for patient prognosis. To further investigate the optimal AR cutoff value associated with long–term prognosis, the ROC curve of AR for predicting recurrent metastatic events was plotted (Figure 2a), and the area under the curve (AUC) was 0.596. Based on the principle of taking the maximum value of the sum of sensitivity and specificity as well as the Youden index, at an AR cutoff value of 65%, the sensitivity was 32.5%; the specificity was 85.1% and the Youden index was 0.168. The ROC curve of AR for the prediction of mortality events was plotted (Figure 2b), and the AUC was 0.644, with a sensitivity of 37.9% and a specificity of 84.3% at an AR cutoff value of 65%. The Kaplan–Meier analysis of patient survival at an AR expression cutoff value of 65% (Figure 2c,d) showed significant differences in survival curves between the two groups (DFS *p* < 0.001; OS *p* < 0.001), with a poor DFS and OS in patients with AR < 65%. In order to further determine whether AR is a prognostic factor independent of ER, we compared the ER expression levels between groups of AR < 65% and ≥65%. The results showed that there was a significant difference in the expression of ER between the two groups. The expression of ER was relatively lower in the subgroup with low AR expression (75.5% vs. 85.5%, *p* < 0.001). This conclusion is consistent with the finding that expression of ER and AR was related in the overall population.

Among the neoadjuvant therapy patients, seven achieved pCR, with one DFS event and no OS events during the follow–up period. A total of 136 patients did not achieve pCR, with 36 DFS events and 10 OS events during the follow–up period. The differences in DFS and OS between the pCR and non–pCR groups were not statistically significant (Table 5).

### 3.4. Prognosis–Related Risk Factors in the Non–pCR Population of Luminal B (HER–2 Negative) Breast Cancer Who Underwent Neoadjuvant Therapy

To further investigate the prognosis–related factors in the neoadjuvant therapy population, DFS multifactorial survival analysis was performed on the non–pCR population of luminal B (HER–2 negative) breast cancer who underwent neoadjuvant therapy. Patients were screened using a backward regression, with age, baseline T–stage, baseline N–stage, histological grade, CNB ER, PR, HER–2, Ki67, AR, AR/ER, AR/PR levels, residual tumor TN stage, residual tumor ER, PR, HER, Ki67, and ΔKi67 as the factors initially included (Table 6). Late baseline T–stage, high AR/ER ratio, and high residual tumor Ki67 levels were risk factors for DFS events. CNB AR, AR/PR, and ΔKi67 were not factors influencing DFS events. The expression of ER is related to the prognosis of breast cancer, so it is necessary to consider the impact of ER on the relationship between AR/ER and prognosis. With the median value of ER expression (90%) as the stratification factor, it was found that AR/ER was variant in the subgroups with different ER expression, and the AR/ER was higher in the group with low ER expression. In the subgroup with ER expression <90%, DFS showed a trend of benefit in the group with AR/ER ≤ 1.00 compared with the group with AR/ER > 1.00, but there was no significant difference between the them (*p* = 0.105). In the ER high expression group, DFS of the population with AR/ER > 1.00 showed a trend of benefit, but there was also no statistical difference (*p* = 0.213). OS multifactorial survival analysis was not performed due to the small number of OS events.

### 3.5. Prognosis–Related AR/ER and Residual Tumor Ki67 Cutoff Values in the Non–pCR Population of Luminal B (HER–2 Negative) Breast Cancer Who Underwent Neoadjuvant Therapy

**Figure 3:**
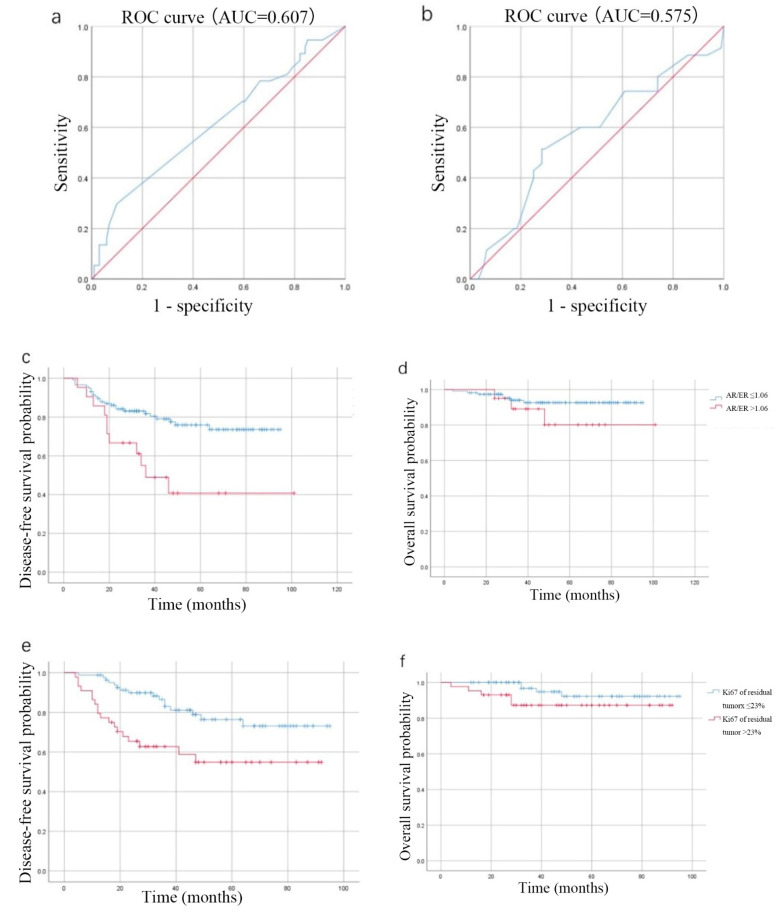
(**a**) ROC curve of AR/ER for predicting DFS events. (**b**) ROC curve of residual tumor Ki67 for predicting DFS events. (**c**) Kaplan–Meier analysis of DFS with an AR/ER cutoff value of 1.06. (**d**) Kaplan–Meier analysis of OS with an AR/ER cutoff value of 1.06. (**e**) Kaplan–Meier analysis of DFS with a residual tumor Ki67 cutoff value of 23%. (**f**) Kaplan–Meier analysis of OS with a residual tumor Ki67 cutoff value of 23%. AR, androgen receptor; ER, estrogen receptor; DFS, disease–free survival; OS, overall survival.

In this part of the study, an AR cutoff value of 65% was selected, AR/ER and AR/PR cutoff values of 1.00 were selected, and a residual tumor Ki67 cutoff value of 20% was selected. Multifactorial survival analysis suggested that AR/ER > 1.00 and residual tumor Ki67 > 20% were risk factors for DFS events. To further investigate the prognosis–related optimal AR/ER cutoff value, the ROC curve of AR/ER for predicting DFS events was plotted (Figure 3a), and the AUC was 0.607. Based on the principle of taking the maximum value of the sum of sensitivity and specificity and Youden index, at an AR/ER cutoff value of 1.06, the sensitivity was 28.6%, the specificity was 90.2% and the Youden index was 0.231. The ROC curve of residual tumor Ki67 for predicting DFS events was plotted (Figure 3b), and the AUC was 0.575, with a sensitivity of 51.4% and specificity of 71.7% at a residual Ki67 cutoff value of 23%. Kaplan–Meier analysis of patient survival was performed using cutoff values of 1.06 for AR/ER and 23% for residual tumor Ki67 (Figure 3c–f). Patients with AR/ER > 1.06 had significantly worse DFS than those with AR/ER ≤ 1.06 (DFS *p* = 0.003) with no significant difference in OS (*p* = 0.222), and patients with residual tumor Ki67 > 23% had significantly worse DFS than patients with residual tumor Ki67 ≤ 23% (*p* = 0.003) with no significant difference in OS (*p* = 0.148).

## 4. Discussion

The 2011 St. Gallen International Expert Consensus proposed classifying breast cancers based on molecular expression markers. Those with positive ER expression and low PR expression or high Ki67 expression were defined as luminal B breast cancer, which accounted for approximately 50–60% of all breast cancers. The systemic treatment of luminal B (HER–2 negative) breast cancer primarily involves endocrine therapy and chemotherapy. The 8–year DFS of patients with luminal B (HER–2 negative) breast cancer is 78.9% [18]. Some patients have a poor response to neoadjuvant chemotherapy and develop recurrence after surgery. The pCR rate of neoadjuvant therapy for this subtype is low, and the relationship with long–term survival is unclear; therefore, large–scale studies are needed to investigate the factors associated with response and prognosis and find sensitive predictors. In this single–centered retrospective study, we evaluated the treatment efficacy and prognosis of patients with luminal B (HER–2 negative) breast cancer in our hospital, investigated the correlation between AR and its associated indicators with patient survival, and aimed to find a prognostic and predictive cutoff value.

AR is a widespread molecular marker across various breast cancer subtypes, and approximately 70–90% of luminal–type breast cancers express AR [19,20]. In ER–negative, AR–positive breast cancers, activated androgen–AR complexes enter the nucleus and bind to androgen response elements in chromatin to induce tumor cell proliferation, whereas in ER–positive, AR–positive breast cancers, activated AR competes with ER to bind estrogen response elements in chromatin and induces apoptosis [21]. A retrospective study suggested that AR expression in luminal–type breast cancer is associated with a better prognosis [22].

A total of 985 patients with luminal B (HER–2 negative) breast cancer were enrolled in this study, with a median follow–up of 42 months. There were a total of 83 DFS events and 29 OS events, and the 3–year DFS was 97.3%, 3–year OS was 98.7%, 5–year DFS was 93.5%, and 5–year OS was 97.4%. In the SOFT trial, the 5–year DFS of patients ranged 84.7–86.6% [23], and in the MINDACT study, the 5–year distant metastasis–free survival (DMFS) was 97.3% in low–risk patients and 90.6% in high–risk patients [24]. In this study, patient survival was similar to those in other studies.

In this study, over 60% of patients had AR expression levels above 90%, and only 2.2% did not express AR. In the literature, the definition of negative and positive AR expression is not consistent, with 1%, 10%, or more, or the Allred score all being used. It is generally accepted that 70–90% of patients with luminal–type breast cancer are positive for AR expression. The high percentage reported in this study may be associated with the difference between Chinese and Western populations and using an AR cutoff value of 1%. The inconsistency of AR cutoff values in the literature affects the comparability of the study results. It informs the need for finding AR prognosis–related cutoff values. The literature reports that male breast cancer patients represent approximately 1% of the patient population, and almost all are ER+ AR+ [25]. A total of seven male breast cancer patients, none of whom underwent neoadjuvant therapy, were included in this study. All had ER expression ≥70% and AR expression ≥90%, which is consistent with the literature. We observed that the overall AR expression levels of patients undergoing neoadjuvant therapy were lower than those not undergoing neoadjuvant therapy. Other characteristics such as age, menopausal status, tumor stage, histological grading, and molecular markers differed significantly, suggesting a significant difference between the neoadjuvant and non–neoadjuvant therapy populations and that the significance of AR may differ between them.

Multifactorial analysis of survival in the overall population suggested that age above 40 years was a protective factor for DFS events. Late T stage, axillary lymph node metastasis, and high histological grade were risk factors for DFS events. In this study, over 60% of patients had at least 90% AR expression levels, and the median AR level of the overall population was 90%. Therefore, a preliminary analysis was performed using an AR expression level of 90% as a cutoff value, and it was observed that the AR expression level correlated with DFS and OS. Further analysis showed that when patients were grouped using an AR of 65% as a cutoff value, there were significant differences in DFS and OS between the two groups, and patients with AR ≤ 65% had significantly worse survival. Recciardelli et al. [26] reviewed 46 studies and included a training cohort (*n* = 219) and a validation cohort (*n* = 418) to determine AR expression cutoff values. Their findings revealed that although most studies selected 1% or 10% as cutoff values, these were not the best cutoff values to differentiate between patients with different outcomes, and they suggested that a positivity cutoff value of 78% had better sensitivity (57.1%) and specificity (75.0%). Patients with AR > 78% had better survival, and those with positive ERα and AR > 78% had the best outcomes. Yang et al. [27] studied 957 breast cancer patients and found that AR ≥ 35% was significantly associated with longer progression–free survival. The population in our study was larger and was a single luminal B (HER–2 negative) breast cancer population; however, the sensitivity of the optimal cutoff point was lower than that of the Recciardeli study. We used 10%, 30% and 78% of AR expression levels as positive cutoff values for survival analysis and tested them in this study cohort. It was found that when taking 10% for analysis, there was no statistical difference in DFS and OS between the two groups of patients in the total luminal B HER2 negative population. When using other AR expression levels as the cutoff value, the populations with AR > 78% or AR > 30% had better DFS and OS, and the results were statistically different. The literature and the present study suggest that AR, unlike ER and PR, should not have a cutoff value of 1% or 10%; however, it should be increased to at least 30%. More studies are still needed to determine the optimal cutoff value. In addition to the heterogeneity of the study population, it is difficult to determine the cutoff value of AR expression level in prognosis prediction. One of the potential factors is that AR may have different biological functions and signal pathways in breast cancer.

In the population of patients who underwent neoadjuvant therapy, 37 DFS events occurred, with a relapse rate of 25.9%. pCR was achieved in only seven individuals in this study, for a pCR rate of 4.9%. Prognostic analysis grouped by pCR or non–pCR suggested that pCR did not correlate with long–term survival. Therefore, further investigation of prognostic correlates in the neoadjuvant therapy population is needed. Currently, there have been few studies on AR in neoadjuvant therapy for breast cancer, and some studies have suggested that AR positivity may be associated with poor neoadjuvant outcomes in TNBC. Witzel et al. [28] reviewed AR expression in the TECHNO and PREPARE clinical trials. Their findings showed that high AR mRNA levels were generally common among HER–2–overexpressing breast cancers and luminal–type breast cancers but extremely rare in TNBC. High AR mRNA levels were associated with low pCR rates but better DFS and OS. The present study did not find any correlation between AR and prognosis in the neoadjuvant therapy population; hence, further discovery of other relevant factors and predictors is needed.

The present study investigated the prognostic, predictive value of AR/ER and AR/PR. Most patients had high AR and ER expression, with a median and mode AR/ER of 1.00. Overall, 14.7% of patients had AR/ER > 1.00, and 31 patients had AR/ER ≥ 2.0, accounting for 3.1% of the study population. There were no statistically significant differences in AR/ER and AR/PR between the neoadjuvant and non–neoadjuvant therapy populations. In the neoadjuvant therapy population, the risk of recurrence was 5.381 times higher in patients with high AR/ER than in those with low AR/ER (95% CI: 1.264–22.91; *p* = 0.023). Further investigation of the cutoff value showed that the optimal AR/ER cutoff value was 1.06, and patients with AR/ER >1.06 accounted for 15.4% of the population who underwent neoadjuvant therapy. Kaplan–Meier analysis suggested that patients with high AR/ER had significantly worse DFS and no significant difference in OS when classified using an AR/ER cutoff value of 1.06. However, the follow–up period of this study was short, and the OS results were incomplete.

The literature shows that AR/ER and AR/PR correlate with endocrine resistance and survival. The study by Cochrane et al. [9] included 192 patients with ER–positive breast cancer who underwent postoperative adjuvant tamoxifen therapy between 1977 and 1993, of whom 48 patients (25%) experienced tamoxifen treatment failure. A high AR/ER ratio (≥2.0) was found in 22 patients, which predicted a fourfold increased risk of tamoxifen treatment failure (HR = 4.43). In addition, this study found that AR/ER was an independent predictor of DFS (HR = 4.04, 95% CI: 1.68–9.69; *p* = 0.002) and disease–specific survival (HR = 2.75, 95% CI: 1.11–6.86; *p* = 0.03). In a study by Rangel et al. [29], 402 patients with ER–positive breast cancer underwent AR, ER, PR, HER–2, and Ki67 testing. ROC curve analysis showed that AR/ER ≥ 2.0 was the cutoff value for distinguishing prognosis and risk factors in patients. A total of 19 patients (6%) had AR/ER ≥ 2.0. These patients exhibited more axillary lymph node metastasis, higher histological grade, lower PR expression levels, and significantly worse disease–free survival and disease–specific survival. Prosigna–PAM50 analysis showed that 63% of patients with high AR/ER (12/19) were categorized with a high risk of recurrence, and 47.4% (9/19) were classified as non–luminal–type. Subsequently, Rangel et al. [30] performed IHC and mRNA analysis to determine AR/ER levels in 47 patients with ER–positive breast cancer and validate them with a public database of 979 patients. They found that ER–positive breast cancer patients with AR/ER ≥ 2 had higher cell proliferation gene expression levels. Most of these patients were of luminal B and HER–2 enriched subtypes, which exhibited a higher degree of proliferation and poorer prognosis. Bronte et al. [31] reviewed a total of 159 ER–positive breast cancer patients between 2000 and 2008 with a median follow–up of 63 months, of whom 89 had data on AR, ER, and survival, and 24 patients with metastasis had pathological findings for primary and metastatic foci. They found that patients with AR/PR ≥ 1.54 in the primary focus had significantly worse OS. In addition, they compared AR/ER in primary and metastatic foci in metastatic patients and found that patients with metastatic foci with AR/ER ≥ 0.90 had better OS, and patients had significantly better OS when AR/ER was maintained at high levels in both primary and metastatic foci. Rajarajan et al. [32] reviewed 270 breast cancer patients with a median follow–up of 72 months. They found that patients with high AR/ER (>1.75, third quartile) had higher circulating testosterone levels and significantly worse DFS, which was a result validated in the Cancer Genome Atlas (TCGA) and the Molecular Taxonomy of Breast Cancer International Consortium (METABRIC) databases. We tested the predictive value of prognosis when the cutoff value of AR/ER was 2.0 and 0.9 in the cohort of our study, but the results were not statistically different.

In luminal–type breast cancers, high AR expression is associated with a good prognosis, and low ER expression is associated with a poor prognosis. Therefore, further investigation is required to determine whether AR/ER is an indirect manifestation of low ER expression. The role of AR in HR–positive breast cancer is controversial, particularly with regard to the relationship between AR and resistance to endocrine therapy. Cochrane et al. [9] found that a high AR/ER ratio was associated with an increased risk of tamoxifen resistance, which was independent of the effects of AR and ER expression levels alone. In MCF7 (ER+ AR+) xenograft tumors, enzalutamide blocked E2–driven tumor proliferation as strongly as tamoxifen, suggesting that high AR/ER may not simply result from low ER expression, but that AR itself does play a role in tumor cell proliferation. The mechanism of endocrine resistance in breast cancer may be the conversion of tumor cells from being estrogen–dependent to androgen–dependent. The mechanism of aromatase inhibitors is to block the conversion of precursors to estrogen and progesterone, causing them to be converted to androgens instead. High androgen levels in patients activate the AR pathway in tumor cells and manifest as endocrine resistance. Indeed, increased anastrozole resistance was found in AR–overexpressing breast cancer patients. In contrast, the AR antagonists bicalutamide and enzalutamide could restore sensitivity to endocrine therapy [33]. In addition to the classic AR signaling pathway, AR also mediates endocrine resistance through other pathways. After knocking down AR in tamoxifen resistant breast cancer cells, tamoxifen sensitivity was restored, which might be the result of upregulation of the estrogen related classical signaling pathway. However, the AR blocker enzalutamide could not completely reproduce the effect of AR knockdown, and the drug could not affect the growth and ER expression of tamoxifen–resistant cells and endocrine–resistant xenograft tumor models [34]. In addition, the interaction between ER and AR has been associated with PR. In ER–positive breast cancer, PR, ERα, and various cofactors interact to form a complex. When ER and PR are activated, high ERβ expression is associated with low invasiveness. This may be because ERα and ERβ form a heterodimer, which reduces the recruitment of regulatory cofactors and thus decreases gene transcription. In ER+ PR+ breast cancers, AR dimers translocate to the nucleus, compete with ERα and PR to bind estrogen–responsive elements, and block ER–mediated signaling pathways. In contrast, in ER+ PR− breast cancers, ERβ acts by downregulating ERα target gene transcription. When PR is absent, AR enhances the effect of ERα gene transcription, producing an oncogenic effect. Clinically, a high AR pathway activity does correlate significantly with worse DFS in patients on endocrine therapy [35]. However, a study by Hickey et al. [8] revealed a contrasting conclusion, proposing that AR exerts an inhibitory effect on the proliferation of HR–positive breast cancer and that AR agonists, rather than antagonists, are potentially effective therapeutic agents for ER–positive breast cancer. The study confirmed that AR competes with ER to bind cofactors and responsive elements in chromatin and block ER–mediated tumor proliferative effects. Thereafter, they established ZR–75–1 xenografts in mice and found that the AR antagonist enzalutamide could only inhibit estradiol (E2)–stimulated tumor proliferation in the short term, whereas the AR agonist dihydrotestosterone (DHT) and the selective androgen receptor modulators (SARM) enobosarm resulted in lasting inhibition of tumor growth, even for over 90 days. In both tamoxifen–resistant ER+ MCF7 xenografts and palbociclib–resistant ER+ MCF7 xenografts, significant tumor suppression was achieved with a combination of CDK4/6 inhibitors and DHT. There may be some heterogeneity in endocrine–resistant breast cancers, and the role of AR remains to be elucidated through in–depth studies.

Ki67 expression levels are predictive of breast cancer prognosis, with higher Ki67 expression levels indicating worse survival. In hormone receptor–positive breast cancer, Ki67 is an important molecular marker to distinguish between luminal A and B breast cancers.

A multifactorial survival analysis of non–pCR patients who underwent neoadjuvant therapy revealed that high residual tumor Ki67 expression was a risk factor for recurrence and metastasis, whereas changes in residual tumor ER, PR, and Ki67 before and after neoadjuvant therapy were not correlated with prognosis. Using the ROC curve, we found that the optimal residual tumor Ki67 cutoff value for distinguishing prognosis was 23%. Non–pCR patients with residual tumor Ki67 < 23% had significantly better survival [36]. Among non–pCR patients in the GeparTrio trial, patients with high residual tumor Ki67 had a higher risk of recurrence and death, whereas the survival of patients with low Ki67 did not differ significantly from that of pCR patients [37]. Due to the low pCR rate of neoadjuvant therapy for HR–positive breast cancer, some researchers have proposed using residual tumor Ki67 level instead of pCR as the index for evaluating response to neoadjuvant endocrine therapy. The preoperative endocrine prognostic index (PEPI) score was derived from the P024 neoadjuvant endocrine therapy clinical trial.. It includes residual tumor size, axillary lymph node status, and Ki67 and ER expression levels. Among them, the cutoff values for Ki67 were 2.7%, 7.3%, 19.7%, and 63.1% [38]. In the ACOSOG Z1031A trial [39], patients with stage II–III ER–positive breast cancer underwent core needle biopsy after 2–4 weeks of neoadjuvant endocrine therapy. Those with Ki67 > 10% were converted to neoadjuvant chemotherapy or immediate surgery, and those with Ki67 < 10% were continued on neoadjuvant endocrine therapy and underwent surgery; only two of the 35 patients who underwent neoadjuvant chemotherapy achieved pCR (5.7%). Furthermore, only 3.7% of patients in the neoadjuvant endocrine therapy group with PEPI = 0 (Ki67 < 2.7%) relapsed after a median follow–up period of 5.5 years, and 14.4% of patients with PEPI > 0 had a relapse event. In the FELINE neoadjuvant endocrine therapy trial [40], PEPI = 0 was the primary study endpoint, with complete cell cycle arrest (Ki67 < 2.7%) as a secondary study endpoint. Similarly, the neoMONARCH neoadjuvant endocrine therapy trial used the magnitude of Ki67 reduction and complete cell cycle arrest (Ki67 < 2.7%) as study endpoints [41].

The advantage of the present study is that it is an early and large population–based, single–center study of the relationship between AR and related indicators and survival among the luminal B breast cancer population in China, and the number of included cases is larger than most foreign studies. This is the first study of the correlation between AR/ER and AR/PR with survival in China. The number of cases far exceeds that of foreign studies related to AR/ER. Moreover, this is the first study of neoadjuvant therapy and AR, AR/ER, and AR/PR in China, and it is also a less studied and novel research topic internationally. The limitations of the study are that it is a single–center, retrospective study that lacks further testing of gene transcription levels; therefore, its results should be validated in a large–sample, prospective study, and gene–level and translational studies are needed to investigate the mechanism.

## 5. Conclusions

Luminal B breast cancer has a relatively good overall prognosis. However, a few patients still exhibit endocrine resistance, early recurrence, and low neoadjuvant chemotherapy pCR rates. Novel prognostic and response–related biomarkers can help screen patients with poor prognoses and may serve as a therapeutic target. In this study, we found that AR influenced the long–term survival of patients with luminal B (HER–2 negative) breast cancer, with an optimal cutoff value of 65%. Patients had significantly worse DFS and OS when AR < 65%. In patients with luminal B (HER–2 negative) breast cancer who underwent neoadjuvant therapy, AR expression level alone was not an influencing factor for prognosis, whereas AR/ER > 1.06 and residual tumor Ki67 > 23% were risk factors for DFS. The study contributes to the early screening of patients with a poor prognosis of luminal B (HER–2 negative) breast cancer and provides a basis for studies on AR–targeted therapies. More extensive and in–depth studies are further needed to address the relationship between luminal B breast cancer and AR.

## Figures and Tables

**Table 1:** Baseline characteristics of the luminal B (HER–2 negative) BC study population.

Baseline Characteristics		Total	Neoadjuvant Chemotherapy	Non–Neoadjuvant Chemotherapy	*p* Value
		985 (100%)	143 (14.5%)	842 (85.5%)	
Sex	Female	978 (99.3%)	143 (100%)	835 (99.2%)	0.602
	Male	7 (0.7%)	0 (0%)	7 (0.8%)	
Age	≤40	93 (9.5%)	28 (19.6%)	65 (7.7%)	<0.001
	>40	891 (90.5%)	115 (80.4%)	776 (92.3%)	
BMI	<24.0	439 (45.8%)	56 (39.7%)	383 (46.8%)	0.118
	≥24.0	520 (54.2%)	85 (60.3%)	435 (53.2%)	
Menopause	No	352 (38.1%)	73 (52.5%)	279 (35.5%)	<0.001
	Yes	573 (61.9%)	66 (47.5%)	507 (64.5%)	
T stage	T0–2	940 (85.8%)	118 (84.9%)	822 (97.6%)	<0.001
	T3–4	31 (14.2%)	11 (15.1%)	20 (2.4%)	
N stage	N0	556 (57.1%)	31 (22.3%)	525 (62.9%)	<0.001
	N+	418 (42.9%)	108 (77.7%)	310 (37.1%)	
Grade	G1–2	750 (77.2%)	83 (59.7%)	667 (80.2%)	<0.001
	G3	221 (22.8%)	56 (40.3%)	165 (19.8%)	
CNB ER	1–10%	21 (2.1%)	9 (6.3%)	12 (1.4%)	<0.001
	11–30%	17 (1.7%)	7 (4.9%)	10 (1.2%)	
	≥30%	947 (96.1%)	127 (88.8%)	820 (97.4%)	
CNB PR	0–10%	286 (29%)	53 (37.1%)	233 (27.7%)	0.032
	11–30%	90 (9.1%)	12 (8.4%)	78 (9.3%)	
	≥30%	609 (61.8%)	78 (54.4%)	531 (63.1%)	
CNB HER–2	IHC −	347 (35.3%)	41 (28.9%)	306 (36.3%)	0.085
	IHC + ~ ++	637 (64.7%)	101 (71.1%)	536 (63.7%)	
CNB AR	0	22 (2.2%)	11 (7.7%)	11 (1.3%)	<0.001
	1–64%	140 (14.2%)	39 (27.3%)	101 (12.0%)	
	65–89%	217 (22.0%)	21 (14.7%)	196 (23.3%)	
	≥90%	606 (61.5%)	72 (50.3%)	534 (63.4%)	
AR/ER	≤1.00	840 (85.3%)	121 (84.6%)	719 (85.4%)	0.809
	>1.00	145 (14.7%)	22 (15.4%)	123 (14.6%)	
AR/PR	≤1.00	442 (44.9%)	59 (41.3%)	383 (45.5%)	0.347
	>1.00	543 (55.1%)	84 (58.7%)	459 (54.5%)	
CNB Ki67	0–10%	54 (5.5%)	1 (0.7%)	53 (6.3%)	<0.001
	11–30%	629 (63.9%)	47 (32.9%)	582 (69.1%)	
	≥30%	302 (30.7%)	95 (66.4%)	207 (24.6%)	
Median follow–up duration (month)		42(4–101)	46 (4–101)	42 (8–79)	
Occurrence of DFS	No	830 (90.9%)	101 (73.2%)	729 (94.1%)	<0.001
	Yes	83 (9.1%)	37 (26.8%)	46 (5.9%)	
Occurrence of OS	No	880 (96.8%)	124 (92.5%)	756 (97.5%)	0.002
	Yes	29 (3.2%)	10 (7.5%)	19 (2.5%)	

**Table 2:** Characteristic of neoadjuvant therapy.

Clinicopathologic Feature		No. of Patients (%)
Neoadjuvant therapy	Chemotherapy	134 (93.7%)
	Anthracycline plus taxane	126 (88.1%)
	Other chemotherapy	8 (5.6%)
	Endocrine therapy	9 (6.3%)
Miller–Payne stage	G1	31 (21.7%)
	G2	10 (7%)
	G3	50 (35%)
	G4	32 (22.4%)
	G5	7 (4.9%)
	Unknown	13 (9.1%)
pCR	No	136 (95.1%)
	Yes	7 (4.9%)
ypT stage	ypT0	7 (4.9%)
	ypT1–2	125 (87.4%)
	ypT3–4	11 (7.7%)
ypN stage	ypN0	46 (32.2%)
	ypN+	96 (67.1%)
	Unknown	1 (0.7%)
ER of residual tumor	1–10%	16 (11.2%)
	11–30%	6 (1.2%)
	≥30%	111 (77.6%)
	pCR/unknown	10 (7.0%)
PR of residual tumor	1–10%	68 (47.6%)
	11–30%	18 (12.6%)
	≥30%	47 (32.9%)
	pCR/unknown	10 (7.0%)
Ki67 of residual tumor	Ki67 ≤ 20%	84 (58.7%)
	Ki67 > 20%	48 (33.6%)
	pCR/ unknown	11 (7.7%)
ΔKi67	Large decrease	31 (21.7%)
	Medium decrease	36 (25.2%)
	Slight decrease/no change	48 (33.6%)
	Increase	15 (10.5%)
	pCR/unknown	13 (9.1%)

**Table 3:** Multivariate analysis of DFS in the luminal B (HER–2 negative) BC study population.

Step	Factors Included		B	SE	Wald	HR (95% CI)	*p* Value
Step 1	Age	≤40				reference	
		>40	−0.696	0.3	5.374	0.499 (0.277–0.898)	0.02
	Baseline T stage	T0–2				reference	
		T3–4	0.923	0.347	7.05	2.516 (1.273–4.971)	0.008
	Baseline N stage	N0				reference	
		N+	0.748	0.272	7.587	2.114 (1.241–3.6)	0.006
	Grade	G1–2				reference	
		G3	0.726	0.262	7.694	2.067 (1.237–3.454)	0.006
	CNB ER	1–10%			1.283	reference	0.526
		11–30%	0.629	0.88	0.51	1.875 (0.334–10.523)	0.475
		≥30%	0.026	0.795	0.001	1.027 (0.216–4.875)	0.973
	CNB PR	0–10%			1.647	reference	0.439
		11–30%	−0.549	0.44	1.558	0.578 (0.244–1.367)	0.212
		≥30%	−0.041	0.327	0.015	0.96 (0.506–1.823)	0.901
	CNB HER–2	IHC −				reference	
		IHC + ~ ++	−0.236	0.254	0.864	0.79 (0.481–1.299)	0.353
	CNB AR	0–89%				reference	
		≥90%	−0.555	0.254	4.767	0.574 (0.349–0.945)	0.029
	AR/ER	≤1.00				reference	
		>1.00	0.326	0.321	1.037	1.386 (0.739–2.598)	0.309
	AR/PR	≤1.00				reference	
		>1.00	0.356	0.306	1.355	1.427 (0.784–2.598)	0.244
	CNB Ki67	0–10%			0.8	reference	0.67
		11–30%	−0.379	0.631	0.36	0.685 (0.199–2.36)	0.549
		≥30%	−0.182	0.653	0.078	0.833 (0.232–2.994)	0.78
	Neoadjuvant therapy	No				reference	
		Yes	0.56	0.282	3.932	1.75 (1.007–3.042)	0.047
Step 7	Age	≤40				reference	
		>40	−0.665	0.29	5.268	0.514 (0.292–0.907)	0.022
	Baseline T stage	T0–2				reference	
		T3–4	0.873	0.329	7.036	2.395 (1.256–4.565)	0.008
	Baseline N stage	N0				reference	
		N+	0.679	0.266	6.52	1.972 (1.171–3.32)	0.011
	Grade	G1–2				reference	
		G3	0.722	0.243	8.848	2.059 (1.279–3.313)	0.003
	CNB AR	0–89%				reference	
		≥90%	−0.555	0.235	5.575	0.574 (0.362–0.91)	0.018
	Neoadjuvant therapy	No				reference	
		Yes	0.675	0.266	6.422	1.964 (1.165–3.31)	0.011

**Table 4:** Multivariate analysis of OS in the luminal B (HER–2 negative) BC study population.

Step	Factors Included		B	SE	Wald	HR (95% CI)	*p* Value
Step 1	Age	≤40					
		>40	0.921	0.796	1.341	2.513 (0.528–11.947)	0.247
	Baseline T stage	T0–2					
		T3–4	1.077	0.599	3.231	2.937 (0.907–9.505)	0.072
	Baseline N stage	N0					
		N+	0.97	0.45	4.638	2.637 (1.091–6.375)	0.031
	Grade	G1–2					
		G3	0.885	0.431	4.221	2.424 (1.042–5.64)	0.04
	CNB ER	1–10%			0.857		0.652
		11–30%	0.619	1.375	0.203	1.857 (0.126–27.486)	0.652
		≥30%	−0.229	1.215	0.035	0.796 (0.074–8.602)	0.851
	CNB PR	0–10%			0.343		0.842
		11–30%	−0.283	0.645	0.193	0.753 (0.213–2.665)	0.66
		≥30%	−0.311	0.61	0.26	0.733 (0.222–2.423)	0.61
	CNB HER–2	IHC −					
		IHC + ~ ++	−0.854	0.405	4.443	0.426 (0.193–0.942)	0.035
	CNB AR	0–89%					
		≥90%	−1.034	0.431	5.755	0.356 (0.153–0.828)	0.016
	AR/ER	≤1.00					
		>1.00	−0.638	0.693	0.845	0.529 (0.136–2.058)	0.358
	AR/PR	≤1.00					
		>1.00	−0.114	0.546	0.043	0.893 (0.306–2.602)	0.835
	CNB Ki67	0–10%			0.053		0.974
		11–30%	−0.059	1.078	0.003	0.943 (0.114–7.804)	0.956
		≥30%	0.044	1.12	0.002	1.045 (0.116–9.383)	0.969
	Neoadjuvant therapy	No					
		Yes	0.013	0.489	0.001	1.013 (0.389–2.639)	0.979
Step 8	Baseline T stage	T0–2					
		T3–4	1.045	0.518	4.065	2.842 (1.03–7.847)	0.044
	Baseline N stage	N0					
		N+	0.921	0.43	4.588	2.511 (1.081–5.831)	0.032
	Grade	G1–2					
		G3	0.872	0.39	4.982	2.391 (1.112–5.139)	0.026
	CNB HER–2	IHC −					
		IHC + ~ ++	−0.825	0.389	4.488	0.438 (0.204–0.94)	0.034
	CNB AR	0–89%					
		≥90%	−1.163	0.401	8.424	0.312 (0.142–0.685)	0.004

**Table 5:** Prognostic analysis of patients in the pCR and non–pCR groups.

Group	DFS	OS
pCR	85.71%	100.00%
non–pCR	73.53%	92.65%
*p* value	0.741	>0.999

**Table 6:** Multivariate analysis of DFS in non–pCR Luminal B (HER–2 negative) group.

Factors Included		B	SE	Wald	HR (95% CI)	*p* Value
Age	≤40				reference	
	>40	−0.411	0.481	0.731	0.663 (0.258–1.701)	0.393
Baseline T stage	T0–2				reference	
	T3–4	1.266	0.571	4.923	3.547 (1.159–10.854)	0.027
Baseline N stage	N0				reference	
	N+	−0.009	0.89	0	0.991 (0.173–5.671)	0.992
Grade	G1–2				reference	
	G3	0.414	0.626	0.436	1.512 (0.443–5.157)	0.509
CNB ER	1–10%			1.273	reference	0.529
	11–30%	1.686	1.498	1.266	5.396 (0.286–101.71)	0.261
	≥30%	1.49	1.674	0.793	4.439 (0.167–118.05)	0.373
CNB PR	0–10%			1.481	reference	0.477
	11–30%	−1.007	0.926	1.183	0.365 (0.06–2.242)	0.277
	≥30%	−0.806	0.801	1.011	0.447 (0.093–2.148)	0.315
CNB HER–2	IHC −				reference	
	IHC + ~ ++	−0.86	0.571	2.269	0.423 (0.138–1.295)	0.132
CNB AR	<65%				reference	
	≥65%	−0.041	0.572	0.005	0.96 (0.313–2.946)	0.943
AR/ER	≤1.00				reference	
	>1.00	1.683	0.739	5.183	5.381 (1.264–22.91)	0.023
AR/PR	≤1.00				reference	
	>1.00	−0.82	0.611	1.8	0.441 (0.133–1.459)	0.18
CNB Ki67	0–10%			2.013	reference	0.365
	11–30%	5.187	85.207	0.004	178.85 (0–6.035 × 10^74^)	0.951
	≥30%	4.31	85.21	0.003	74.467 (0–2.528 × 10^74^)	0.96
ypT stage	T0–2				reference	
	T3–4	0.969	0.884	1.201	2.637 (0.466–14.925)	0.273
ypN stage	ypN0				reference	
	ypN+	−0.046	0.761	0.004	0.955 (0.215–4.241)	0.951
ER of residual tumor	1–10%			2.709	reference	0.258
	11–30%	−1.385	1.556	0.792	0.25 (0.012–5.283)	0.373
	≥30%	0.695	1.181	0.346	2.004 (0.198–20.302)	0.556
PR of residual tumor	1–10%			0.614	reference	0.736
	11–30%	−0.08	0.812	0.01	0.923 (0.188–4.532)	0.921
	≥30%	0.395	0.625	0.4	1.485 (0.436–5.052)	0.527
HER–2 of residual tumor	−				reference	
	+ ~ ++	0.241	0.369	0.427	1.273 (0.617–2.626)	0.513
Ki67 of residual tumor	Ki67 ≤ 20%				reference	
	Ki67 > 20%	1.607	0.676	5.658	4.988 (1.327–18.748)	0.017
ΔKi67	Large decrease			5.641	reference	0.13
	Medium decrease	0.577	0.834	0.478	1.78 (0.347–9.132)	0.489
	Slight decrease/no change	−0.079	0.89	0.008	0.924 (0.162–5.286)	0.929
	Increase	−2.241	1.439	2.425	0.106 (0.006–1.785)	0.119

## Data Availability

The datasets generated and analyzed during the current study are not publicly available due to patients’ individual privacy but are available from the corresponding author on reasonable request.
